# Blood and salivary lactate responses to active rest following circuit exercise

**DOI:** 10.3389/fphys.2025.1534668

**Published:** 2025-05-15

**Authors:** Chihiro Kojima, Takuma Morishima, Reita Ito, Sosuke Yasunaga, Takumi Shimogaki, Takeshi Hashimoto, Tadao Isaka, Motoyuki Iemitsu

**Affiliations:** ^1^ Research Organization of Science and Technology, Ritsumeikan University, Shiga, Japan; ^2^ Faculty of Liberal Arts and Sciences, Chukyo University, Nagoya, Japan; ^3^ Faculty of Sport and Health Science, Ritsumeikan University, Shiga, Japan

**Keywords:** active recovery, circuit exercise, lactate, testing, salivary

## Abstract

The present study investigated blood and salivary lactate concentrations with or without active recovery following intensive exercise. Seven men and four women were randomly assigned to two trials consisting of a control (CON) trial and an active recovery (AR) trial separated by at least 1 week. In both trials, participants performed total 30 min of circuit exercise for lower extremities. Afterwards, in the AR trial, participants completed 15 min of light cycling exercise; in the CON trial, participants remained seated during postexercise. Blood and salivary concentrations were measured before and immediately after the circuit exercise, immediately after each trial, and the next morning, using a portable device. Blood lactate concentrations increased significantly immediately after circuit exercise in both trials (AR: 11.9 ± 2.9 mmol/L, CON: 13.4 ± 3.0 mmol/L, *P* < 0.05), and a significant difference was observed between trials, immediately after each trial (AR: 4.2 ± 1.4 mmol/L, CON: 6.9 ± 2.8 mmol/L, *P* < 0.05). In addition, both trials showed salivary lactate concentrations increased significantly following circuit exercise (AR trial: 4.4 ± 3.0 mmol/L, CON trial: 5.4 ± 3.9 mmol/L, *P* < 0.05), and there was a significant difference between trials after the intervention (AR trial: 0.8 ± 0.4 mmol/L, CON trial: 3.1 ± 2.0 mmol/L, *P* = 0.007). In addition, there was a significant correlation between blood and salivary lactate concentrations during the postexercise period (r = 0.856, *P* < 0.001). Thus, salivary lactate concentrations may reflect relative changes in blood lactate concentrations during the postexercise period with active recovery following intensive exercise.

## 1 Introduction

Blood lactate concentration can be used to evaluate the state of energy supply in each organ. Therefore, the time-course of evaluation of blood lactate concentration during and/or after exercise reflects the enhancement of glycolysis for anaerobic energy metabolism in muscles. In addition, an evaluation of the blood lactate concentration during and after exercise is also useful for evaluating anaerobic and aerobic exercise capacities and exercise load. Because blood lactate concentrations are easily measured with portable devices (e.g., Lactate Pro 2: ARKRAY Co. Ltd., Japan), the evaluation of blood lactate concentration has been accepted in sports science fields as a powerful tool. However, the assessment of blood lactate concentration with acute exercise requires repeated invasive sampling of small amounts of blood, which may induce greater physical burdens among subjects and ethical issues. Thus, noninvasive techniques are needed in the future.

Given that blood lactate concentration is determined by the balance between production and removal (Stainsby and Brooks, 1990), it is necessary that alternative strategies to invasive measurement methods are sensitive to various situations. Saliva is produced by transferring components from the blood through the salivary glands (Drobitch and Svensson, 1992; Edgar, 1992); thus, saliva is likely to contain lactic acid that has accumulated in the blood. Several studies have examined changes in both blood and salivary lactate concentrations immediately after acute exercise using incremental pedaling exercise (Oliveira et al., 2015; Segura et al., 1996), maximal running on a treadmill (Tékus et al., 2012) and vertical squat jump (Okano et al., 2021) and, in field-based studies, during actual 30-km running (Santos et al., 2006) or immediately after 400-m running on a track (Ohkuwa et al., 1995). These findings suggest that the assessment of salivary lactate concentrations has the potential to be a surrogate for blood lactate concentrations during and after exercise. However, although the time-course changes in salivary lactate concentration following exercise have been gradually clarified, it remains unclear how salivary lactate concentration changes during postexercise period with certain interventions. To utilize salivary lactate concentration in the field, it is necessary to be able to monitor changes in lactate concentration during postexercise recovery with specific strategies to remove lactate quickly (e.g., light exercise and nutritional intake). Previous studies offer only limited evidence of successful application.

Some studies have proposed active recovery as one of the ways to remove lactate rapidly from active muscles (McAinch et al., 2004; Bangsbo et al., 1985; Ahmaidi et al., 1996). For instance, Corder et al. (JAP et al., 2000) demonstrated that active recovery with pedaling exercise following resistance exercise resulted in a rapid reduction in blood lactate concentrations, compared with passive recovery. Greenwood et al. (2008) suggested that the intensive swimming exercise-induced increase in blood lactate concentration was rapidly removed with active recovery with swimming exercise. The mechanisms by which active recovery rapidly reduces blood lactate have been indicated to involve hepatic oxidation, increased myocardial utilization, and oxidation as fuel in working muscles (Gisolfi et al., 1966). As such, although active recovery contributes to lactate clearance, the assessment requires continuous monitoring of lactate levels remaining *in vivo* during postexercise. However, it remains unclear whether salivary lactate concentrations reflect changes in blood lactate clearance with active recovery after exercise. Therefore, the present study investigated the blood and salivary lactate concentrations during the postexercise period, with or without active recovery, following intensive exercise.

## 2 Methods

### 2.1 Participants

Twelve heathy participants were recruited in the present study. However, a participant dropped out due to a medical issue. Therefore, the study participants included seven men and four women (age: 20.6 ± 0.9 years, height: 170.3 ± 8.3 cm, weight: 62.5 ± 7.2 kg). All participants were healthy individuals with non-smokers and no known cardiometabolic diseases (Table 1). The participants were informed of the study’s purpose, experimental procedure, and potential risks. Written informed consent was obtained from all participants. This study was approved by Ritsumeikan University, Japan (BKC-LSMH-2023-061) and conducted in accordance with the relevant guidelines and regulations or declaration of Helsinki.

**TABLE 1:** Characteristics of participants in the present study.

	Male (n = 7)	Female (n = 4)
Height (cm)	175.1 ± 5.7	161.8 ± 5.7
Weight (kg)	65.0 ± 7.8	58.1 ± 5.4
$\overset{\cdot }{\text{V}}$ O_2max_ (mL/min/kg)	50.6 ± 5.2	37.5 ± 4.6
1RM for leg extension	148.0 ± 24.8	94.3 ± 3.5
1RM for leg curl	92.0 ± 13.3	59.3 ± 3.5

Values are means ± SD. 1RM: one-repetition maximum.

### 2.2 Experimental design

The present study used a randomized cross-over design. All participants completed two trials, consisting of a control trial (CON trial) and an active recovery trial (AR trial). Each trial was separated by at least 1 week. In both trials, participants performed six sets of 5-min circuit exercise (total 30 min) comprised of three exercises for the lower extremities and rest. After circuit exercise, the participants in the AR trial conducted 15 min of light pedaling exercise at around 10 min after the circuit exercise period, under a load that allowed the heart rate to reach 120 bpm; in the CON trial, the participants remained seated and resting for an identical period of time (15 min). Blood and salivary samples were obtained at the timings of before and after completing the circuit exercise (denoted hereinafter as *Pre-exercise* and *After exercise*, respectively), after active recovery or rest (as *After intervention*), and the next morning (as *After 24 h*). In addition, subjective feeling of fatigue and the counter movement jump (CMJ) height were evaluated at the identical timing of blood and salivary sampling as an indication of recovery from the intensive exercise. Water intake (1,200 mL) and meal intake (1,068 kcal) during the experimental period were controlled during the two trials.

### 2.3 Circuit exercise protocol (fatigue protocol)

Three hours before starting the circuit exercise, participants consumed carbohydrates (180 kcal: in jelly, Morinaga & Co., Ltd., Tokyo, Japan), and their intake was controlled between trials. As the fatigue protocol, participants performed 30 min of circuit exercise in both trials. The exercise consisted of six sets, each lasting 5 min. The sets comprised two resistance exercises for the lower extremities (each 1 min, thus 2 min total), pedaling exercise (2 min), and rest (1 min). As resistance exercises, leg extensions and leg curls were performed at 30% of individual one repetition maximum (1RM) each using weight stack machines (Life Fitness, Tokyo. Japan). Participants were asked to lift and lower the weight in 1-s and 2-s intervals, respectively [actual load: 38.5 ± 9.5 kg (leg extension), 24.0 ± 5.3 kg (leg curl)]. In addition, participants conducted pedaling exercise on a cycling ergometer (828E, Monark, Stockholm, Sweden) for 2 min under a load of 215.6 ± 44.3 W to elicit −70% of maximal oxygen uptake ( $\overset{\cdot }{\text{V}}$ O_2max_), with a pedaling frequency of 80 rpm. The intensities of resistance and cycling exercises were adjusted as necessary to ensure the completion of each set. The adjusting of the intensity was kept the same between trials. The protocol was determined by our preliminary experiments.

### 2.4 Recovery protocol

In the AR trial, participants performed 15 min of pedaling exercise on a cycling ergometer (828E, Monark), under an individual load of 76.5 ± 28.1 W that allowed the heart rate to reach 120 bpm (de Azevedo Franke et al., 2021). The exercise intensity was determined from the regression formula between exercise load and heart rate during the $\overset{\cdot }{\text{V}}$ O_2max_ test for each participant. The pedaling frequency was set to 60 rpm. In the CON trial, participants remained seated for a period of 15 min, which was identical to the AR period in the AR trial.

### 2.5 Measurements

#### 2.5.1 Maximal oxygen uptake (VO_2max_)

$\overset{\cdot }{\text{V}}$ O_2max_ was assessed using an incremental pedaling test via a cycling ergometer (828E, Monark), to determine exercise intensity during subsequent main trials. The test began at 74 or 88 W, and the load increased progressively in 15-W increments until voluntary exhaustion. The test was terminated when the participants failed to maintain the prescribed pedaling frequency of 60 rpm or reached the V^.^O_2_ plateau. Respiratory gases were collected during exercise and analyzed using an automatic gas analyzer (AE310, Minato Medical Science Co., Ltd., Tokyo, Japan), to evaluate V^.^O_2_, carbon dioxide, and ventilatory volume. Data were averaged every 30 s.

#### 2.5.2 One-repetition maximum

1RM for the leg curl and leg extension exercises was also evaluated to determine exercise intensity during subsequent main trials. Before the test, participants warmed up with two exercises at 10 repetitions each. The intensity was increased progressively, until the participant failed to perform the lift fully, as described earlier. The series of measurements were conducted using weight stack machines (Life Fitness, Tokyo, Japan).

#### 2.5.3 Blood lactate concentrations

Blood samples were collected before and after completing the circuit exercise (*Pre-exercise* and *After exercise*, respectively), after active recovery or rest (*After intervention*), and the next morning (*After 24 h*) from the fingertip. From these samples, lactate concentrations were measured using a lactate analyzer (Lactate Pro2, ARKRAY Co., Ltd., Kyoto, Japan) immediately after blood sampling.

#### 2.5.4 Salivary lactate concentrations

Salivary samples were collected at the same time as blood sample collection. According to our previous study (Uchino et al., 2024), the participants rinsed their mouths with distilled water for 30 s, three times before exercise, followed by saliva sample collection. Saliva production was stimulated by chewing a piece of paraffin wax (B.S.A paraffin wax, B.S.A, Aichi, Japan) for 1 min at a frequency of 1 chew/s. The collected saliva was separated from the paraffin wax by centrifugation at 4°C at 1,500 × g for 5 min and stored at −20°C until analysis. Salivary lactate concentrations were measured using a lactate analyzer (Lactate Pro2, ARKRAY Co., Ltd.), whose was already reported the validity to measure the salivary lactate concentration in a previous study (Okano et al., 2021).

#### 2.5.5 Fatigue

Subjective feeling of fatigue was evaluated at the timings of *Pre-exercise*, *After exercise*, *After intervention*, and *After 24 h* using a 100-mm visual analogue scale.

#### 2.5.6 Counter movement jump

Maximal CMJ height was measured at the four timings (*Pre-exercise*, *After exercise*, *After intervention*, and *After 24 h*) using a mat (Multi Jump Tester Ⅱ, Q’sfix Co. Ltd., Japan) that was connected to a computer. Participants were instructed to perform a jump as high as possible without upper-limb effects.

### 2.6 Statistical analysis

Data are expressed as the mean ± standard deviation (SD). The sample size was determined via a power analysis using customized computer software (GPOWER Version 3.1.9, University of Dusseldorf, Germany). The power analysis suggested a sample size of 12, for an expected power of 0.8 with an alpha level of *p* ≤ 0.05. All data were tested for normality using Kolmogorov-Smirnov test. Only the salivary lactate concentration data failed to meet the normality assumption. Time-course changes in blood lactate concentration, fatigue and CMJ height were compared using two-way repeated measures ANOVA to determine interaction between time and trial and the main effects (time and/or trial). When the ANOVA revealed significant effects, we performed post-hoc pairwise comparisons with Bonferroni adjustment to control for multiple comparisons. Because salivary lactate concentration was not normally distributed, we used the nonparametric Friedman test to compare conditions over time. When the Friedman test indicated a significant difference, we conducted pairwise comparisons between time points using Wilcoxon’s signed-rank test. The relationship between blood and saliva lactate concentrations was determined using Spearman`s rank correlation coefficient. Statistical significance was accepted as a *P*-value <0.05.

## 3 Results

### 3.1 Subjective feeling of fatigue

There was no significant difference between the CON and AR trials *Pre-exercise* (baseline) in fatigue. Two-way ANOVA revealed time as one of the main effects (*P* < 0.001), although there was no significant interaction (*P* = 0.702), as well as the main effect of trial (CON vs. AR) (*P* = 0.790, Table 2). Subjective feeling of fatigue was significantly higher *After exercise* and *After intervention* (*P* < 0.05) relative to that *Pre-exercise*.

**TABLE 2:** Time-course of changes in subjective feeling of fatigue.

(mm)	*Pre-exercise*	*After exercise*	*After intervention*	*After 24 h*	Interaction	Main effect	
						Time	Trial
AR trial	32.3 ± 20.5	76.3 ± 15.1	49.5 ± 15.8	37.7 ± 20.6	0.702	<0.001	0.790
CON trial	34.5 ± 15.1	75.8 ± 12.0	48.4 ± 17.8	42.5 ± 22.5			

Values are means ± SD.

### 3.2 CMJ height

During the Pre-exercise period, no significant difference was observed between the CON and AR trials in CMJ height. Although a significant main effect of time was observed (*P* = 0.002), there was no significant interaction (*P* = 0.319) or main effect of trial (P = 0.073, Table 3). CMJ height was significantly lower After exercise and After 24 h (*P* < 0.05) relative to that recorded for Pre-exercise.

**TABLE 3:** Time-course of changes in CMJ height.

(cm)	*Pre-exercise*	*After exercise*	*After intervention*	*After 24 h*	Interaction	Main effect	
						Time	Trial
AR trial	34.3 ± 9.7	29.4 ± 9.3	34.7 ± 10.1	32.2 ± 9.7	0.319	0.002	0.073
CON trial	33.8 ± 9.2	29.1 ± 10.0	32.3 ± 9.2	31.8 ± 8.9			

Values are means ± SD.

### 3.3 Blood and salivary lactate concentrations

**FIGURE 1:**
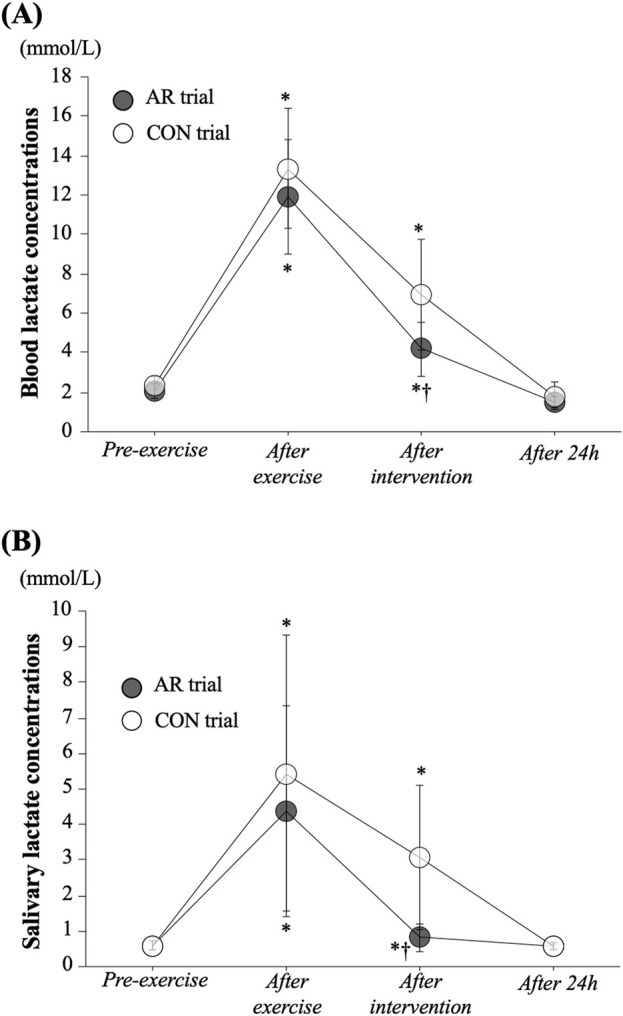
Time-course changes in blood **(A)** and salivary **(B)** lactate concentrations. Values represent mean ± standard deviation. *: *P* < 0.05 vs. Pre, †: *P* < 0.05 vs. CON trial.

Figures 1A,B show the time-course of changes in blood lactate concentrations and salivary lactate concentrations, respectively. No significant difference between trials was observed *Pre-exercise* (baseline) for either. For the blood lactate concentrations, a significant interaction (*P* = 0.019) and the main effects of time (*P* < 0.001) and trial (*P* = 0.024) were observed. In both the CON and AR trials, blood lactate concentrations were increased significantly with circuit exercise (AR: 11.9 ± 2.9 mmol/L, CON: 13.4 ± 3.0 mmol/L) and the significant elevations remained until the *After intervention* period (*P* < 0.05). However, blood lactate concentrations *After intervention* were significantly lower in the AR trial than that in the CON trial (AR: 4.2 ± 1.4 mmol/L, CON: 6.9 ± 2.8 mmol/L, *P* < 0.05). For the salivary lactate concentration, both trials showed significant elevation at *After exercise* and *After intervention* (AR trial: χ^2^ (Edgar, 1992) = 20.43, *P* < 0.001, CON trial: χ^2^ (Edgar, 1992) = 26.57, *P* < 0.001). Also, at *After intervention*, salivary lactate concentration in AR trial was significantly lower than that in the CON trial (AR trial: 0.8 ± 0.4 mmol/L, CON trial: 3.1 ± 2.0 mmol/L, *P* = 0.007).

**FIGURE 2:**
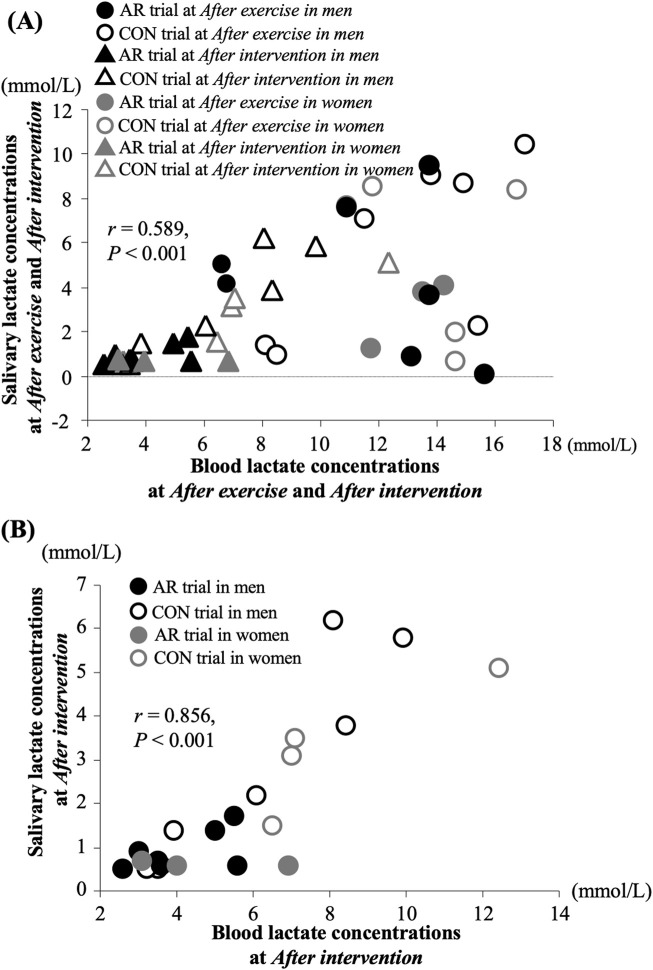
Relationship between blood and salivary lactate concentrations *After exercise* and *After intervention* **(A)** and *After intervention* **(B)**.

The present study observed a significant relationship between blood and salivary lactate concentrations *After exercise* and *After intervention* (Figure 2A, r = 0.589, *P* < 0.001). Furthermore, there was also a significant relationship between blood and salivary lactate concentrations *After intervention* (Figure 2B, r = 0.789, *P* < 0.001).

## 4 Discussion

The present study investigated the relationship between blood and salivary concentrations with active recovery following intensive exercise. The results demonstrated for the first time that changes in salivary lactate concentration with active recovery after acute exercise can be used to detect responses similar to those in blood lactate levels.

Active recovery following intensive exercise has been well utilized as a strategy to reduce lactate rapidly in the blood (McAinch et al., 2004; Bangsbo et al., 1985; Ahmaidi et al., 1996; Jap et al., 2000; Greenwood et al., 2008). The present study investigated blood and salivary lactate concentrations following intensive exercise, with or without active recovery at the individual exercise intensity at which the heart rate reached 120 bpm (35.9% ± 8.3% of $\overset{\cdot }{\text{V}}$ O_2max_). A significant reduction in the blood lactate concentration was observed after active recovery (64.0% ± 9.0% of reduction). Although significant differences between trials in subjective fatigue and CMJ height were not observed (Pernigoni et al., 2024; Taipale et al., 2018), the clearance effects of active recovery on blood lactate concentration were evident, in accordance with previous studies (Bangsbo et al., 1985; Ahmaidi et al., 1996).

Several previous studies have investigated blood and salivary lactate concentrations during the postexercise period, indicating parallel changes between blood and salivary lactate concentrations (Oliveira et al., 2015; Okano et al., 2021; Ohkuwa et al., 1995). Therefore, these findings suggest that assessing salivary lactate concentration after exercise may be meaningful for evaluating the magnitude of recovery after exercise. However, the validity of measuring salivary lactate concentration during postexercise recovery, with and without active recovery, remains unclear. The present study is the first to attempt to clarify the changes in salivary lactate concentration with active recovery after intensive exercise. In the present study, a significant relationship between blood and salivary lactate concentrations in both timings of *After exercise* and *After intervention* suggests that salivary lactate concentration reflects exercise-induced changes in blood lactate concentrations (Figure 2A). Furthermore, most of the individual data from the AR trial showed lower values compared to those in the CON trial (Figure 2B), suggesting that the elimination effects of active recovery on lactate concentration were observed in both the blood and saliva. Salivary lactate concentration exhibited small changes during the postexercise period with active recovery, with sensitivity comparable to that of blood lactate concentration.

The Lactate Pro 2 is a powerful device for measuring lactate concentration simply and instantly; however, careful attention must be paid to the following in the evaluation of salivary lactate concentration with exercise. The present study observed that the mean values of salivary lactate concentration were lower than blood lactate concentration, in all of the results. A previous study (Okano et al., 2021) that utilized the same device as the present study for measuring blood and salivary lactate concentrations (Lactate Pro2, ARKRAY Co., Ltd.) indicated that the device has a measuring range of 0.5–25.0 mmol/L and that the inability to detect smaller values, i.e., baseline at rest, may be a limitation for measuring salivary lactate concentration with the device. Moreover, in a few participants, an exercise-induced increase in salivary lactate concentration was not clearly observed (3 of 11 participants had concentration levels 1 SD below the average value in each trial, respectively) in the present study. Such an observation was not common among the participants and its implication for detecting an exercise-induced increase in salivary lactate concentration remains unclear in the present study. Further investigations are required to determine the validity of using the Lactate Pro 2 to measure salivary lactate concentration.

This study had several limitations. First, the timings of evaluating salivary lactate concentration were less frequent than in the previous studies. Further salivary samples with active recovery more repeatedly (e.g., 3, 5, 7 and 10 min following exercise) are required to obtain more information regarding changes in salivary lactate concentration with active recovery. Second, the present study did not take into account the salivary volume in reference to a previous study (Okano et al., 2021). Because there was no significant difference between trials in salivary volume following active recovery or seated rest in the present study (AR trial: 3.04 ± 0.13 mL, CON trial: 2.84 ± 1.1 mL, *P* = 0.377), we believe that the effects of changes in salivary volume with active recovery were relatively small. In addition, number of subjects were not enough to develop the applicability of the salivary lactate concentration in the present study. And, the present study recruited less females than males, and the influences of difference in sex and muscle mass were not identified. Therefore, the measurements are required for various populations (e.g., age, sex and fitness levels). Furthermore, because detail mechanisms for lactate recovery kinetics between blood and salivary remains unclear in the present study, future investigations are needed to evaluate the related parameters during postexercise period (e.g., oxygen uptake and acid-base balance). Lastly, salivary lactate concentrations were evaluated following biochemical treatment in the present study. Considering the practical utilization, it is necessary to detect the values more rapidly and easily. We have plan to measure salivary lactate concentrations with a potable devise soon after obtaining samples, and the limitation can be resolved in a future study.

In conclusion, salivary lactate concentration may reflect changes in blood lactate concentration during the postexercise period with active recovery, after intensive exercise. The present results are expected to contribute to the evaluation of various situations involving athletes, those recovering from injury, and older adults.

## Data Availability

The raw data supporting the conclusions of this article will be made available by the authors, without undue reservation.
